# Unraveling the Intricate Nexus of Molecular Mechanisms Governing Rice Root Development: OsMPK3/6 and Auxin-Cytokinin Interplay

**DOI:** 10.1371/journal.pone.0123620

**Published:** 2015-04-09

**Authors:** Pallavi Singh, Tapan Kumar Mohanta, Alok Krishna Sinha

**Affiliations:** National Institute of Plant Genome Research, Aruna Asaf Ali Marg, New Delhi, 110067, India; CSIR-National Botanical Research Institute, INDIA

## Abstract

The root system is an imperative component of a plant, involved in water and nutrient acquisition from the soil. Any subtle change in the root system may lead to drastic changes in plant productivity. Both auxin and cytokinin are implicated in regulating various root developmental aspects. One of the major signaling cascades facilitating various hormonal and developmental allocations is the Mitogen Activated Protein Kinase (MAPK) cascade. Innumerable efforts have been made to unravel the complex nexus involved in rice root development. In spite of a plethora of studies, a comprehensive study aiming to decipher the plausible cross-talk of MAPK signaling module with auxin and cytokinin signaling components in rice is missing. In the present study, extensive phenomics analysis of different stages of rice roots; transcript profiling by qRT-PCR of entire gene family of MAPK, MAPKK and PIN genes; as well as protein level and activity of potential MAPKs was investigated using western and immuno kinase assays both on auxin and cytokinin treatment. The above study led to the identification of various novel rice root specific phenotypic traits by using GiA roots software framework. High expression profile of OsMPK3/6, OsMKK4/5 and OsPIN 1b/9 and their marked transcript level modulation in response to both auxin and cytokinin was observed. Finally, the protein levels and activity assay further substantiated our present findings. Thus, OsMPK3/6-OsMKK4/5 module is elucidated as the putative, key player in auxin-cytokinin interaction augmenting their role by differentially regulating the expression patterns of OsPIN 1b/9 in root development in rice.

## Introduction

The daunting issue of nutritional and food security has resulted in a quest among researchers to improve the rice landraces, with a particular focus on rice roots. The rice root system has emerged as a target for breeders to improve the ability of the plant to exploit the mineral and water resources. The root system is an indispensable component for plant growth and survival necessitating essential functions during plant development, including anchorage and acquisition of water and nutrients from the soil. The structure of root system is governed by endogenous genetic programs as well as by external environmental cues, including a number of biotic and abiotic stresses [1]. Any subtle change in the root system architecture will lead to drastic effects on crop yields. The development of root system and determination of its architectural parameters is an important agronomic trait [2]. Breeding for root architecture involves identifying the genetic determinants of root development which in turn represent a bottleneck for deciphering the converging point of various signaling parameters [3].

Hormone signaling plays diverse and critical roles during root development. Indole-3-acetic acid (IAA) is the predominant form of auxin in plants, while cytokinins are adenine derivatives. Cytokinins have been implicated in regulating many aspects of development including regulation of root growth, root architecture and vascular development whereas auxin promotes root development and induces vascular differentiation. Both auxin and cytokinins regulate root gravitropism [4,5]. Auxin is actively transported between cells to create local maxima necessary for mediating numerous developmental processes. The directional transport is mostly controlled by the sub-cellular polarity of PIN proteins. PIN proteins are auxin efflux carriers which mediate auxin acropetal flow to root tip through the central vasculature and basipetal flow through the epidermis. Eight PIN genes have been identified from *Arabidopsis* genome while their number constitute to twelve in rice. [6–8]. Cytokinin is also reported to modify the expression of multiple PIN genes to regulate polar auxin transport, auxin levels within specific cells and therefore meristem size [5]. Cytokinin–auxin antagonistic interactions in controlling root development is well established [9,10]. The cytokinin transcription network is regulated by a negative feedback loop to control its response to root development via auxin signal transduction pathway [11]. Considering the breadth of functions auxin and cytokinin orchestrate, multiple signaling pathways may regulate root developmental processes. One of the major signaling cascade facilitating various hormonal, environmental stress and developmental allocation is the Mitogen Activated Protein Kinase (MAPK) cascade, a three-tier phospho-relay signaling module that is evolutionarily conserved across eukaryotes. Eukaryotic MAPK cascade transduces environmental and developmental cues into intracellular responses and plays a central role as the controller of gene expression [12–14]. In a simulated model, this canonical network is stimulated by activation of Mitogen Activated Protein Kinase Kinase Kinase (MAP3K/MAPKKK/MEKK/MKKK), which phosphorylates and activates the downstream Mitogen Activated Protein Kinase Kinase (MAPKK/MEKs/MKK) which in turn activate Mitogen Activated Protein Kinase (MAPK/MPK) upon phosphorylation [15,16]. The activated MAPK causes the activation of various transcription factors and cytosolic proteins that can trigger a myriad of transcriptomic, cellular and physiological responses [17]. Genome sequencing of rice identified 15 MAPK, 8 MAPKK and around 75 MAPKKK in rice [18,19].

The involvement of various MAPK in regulation of root development has been demonstrated, predominantly only in the model research eudicot plant, *Arabidopsis*. The differential spatial and temporal expression as well as localization of MPK6 is involved in cell division plane control and root development [20] in *Arabidopsis*. In a recent report, MPK6 was also implicated to cause repression in primary root growth and development as was evident by increment in primary root growth by enhanced cell production and cell elongation in mutant lines [21]. A coherent role of MAPK cascade component and auxin signaling has also been reported. MAPK kinase-7 (MKK7) has been involved in the control of root system architecture through negative regulation of polar auxin transport while, MKK6 and MPK13 were involved in lateral root development [22–24]. MPK12 acts as a negative regulator of auxin signaling and also a substrate of indole-3-butyric acid-response 5 (IBR5), which encodes a dual-specificity MAPK phosphatase. It has been demonstrated that IBR5 phosphatase inactivates MPK12 by dephosphorylating, which in turn leads to the suppression of MPK12 and up-regulation of auxin-responsive genes involved in root development [25].

Although, there have been a repertoire of studies speculating the role of MAPK cascade in root development, our current understanding of MAPK signaling module regulating root development is in naivety. To date, there is no report of a comprehensive analysis of the involvement of complete MAPK cascade components in rice root development. Hence, it is imperative to unravel the intricacies of MAPK cascade and its plausible cross-talk with auxin and cytokinin signaling components, with a particular focus on crop plant and a model monocot plant, rice. The current study aims to decipher the missing links in our present understanding of MAPK and rice root development. A high throughput phenomics analysis of various early stages of rice roots under auxin and cytokinin treatment was undertaken. To get a deeper insight at the molecular level and to identify the potential MAPK signaling component involved during root development, transcript profiling by qRT-PCR of the entire gene family of MAPK and MAPKK in rice was performed. Auxin and cytokinin play a pivotal role during root development. Expression of PIN genes are implicated in differential auxin translocation and is also regulated by cytokinin thus, transcript profiling of PIN genes were carried out on the roots grown in the presence of these phytohormones. Further, the protein level and activity of potential MAPKs was investigated using western and immuno kinase assays, respectively. The above study led to the identification of novel rice root specific phenotypic traits in response to auxin and cytokinin at different developmental stages using GiA roots software framework. High expression profile of OsMPK3/6, OsMKK4/5 and OsPIN 1b/9 and their marked transcript level modulation in response to both auxin and cytokinin was observed. Finally, the protein levels and activity assay further substantiated our present findings. Thus, OsMPK3/6, OsMKK4/5 were elucidated as the putative, key players in auxin-cytokinin interaction augmenting their role by differentially regulating the expression patterns of OsPIN 1b/9 in root development in rice.

## Materials and Methods

### Plant material and hormone treatments

*Oryza sativa* L. indica cultivar group var Pusa Basmati 1 was used in the present study. Plants were grown on ½ MS (Murashige and Skoog) media in sterile glass bottles in growth chamber at 28°C with 16/8 day light condition up to 4 weeks. Exogenous hormone treatments of auxin and cytokinin were given by germinating and growing plants in 1 μM and 5 μM of Indole-3-Acetic Acid (IAA) and 6-Benzylaminopurine (BAP) in ½ MS media, respectively. Roots of 7, 14, 21 and 28 days old rice seedlings were used for the experiments.

### GiA Roots Analysis

The roots of 7, 14, 21 and 28 days old rice seedlings were imaged by rotating for 360° with 20 snap shots at an interval of 18° each. These images were further imported into GiA Roots Software Framework [26] and analyzed for various phenotypic parameters like average root width (diameter), network bushiness, number of connected components, network depth, ellipse axes ratio, network length distribution, major ellipse axis, maximum number of roots, network width, median number of roots, minor ellipse axis, network area, network convex area, network perimeter, network solidity, specific root length, network surface area, network length, network volume and network width to depth ratio.

### qRT-PCR Analyses

The total RNA was extracted from hormone treated as well as untreated roots by Trizol reagent (Sigma Aldrich, USA) according to manufactures protocol. RNA was treated with 10 units of RNase free DNase 1 (Fermentas). 2 μg of total RNA was subjected to first strand cDNA synthesis using RevertAid H Minus First Strand cDNA synthesis kit (Fermentas) as per manufacturer’s protocol using oligo dT primers. The qRT-PCR was carried out in 384 well plate ABI Prism 7000 sequence detection system (Applied Biosystems, City, CA), as has been described previously [27,28]. The relative gene expression was determined based on the 2^−ΔΔCT^ method [29]. Primer pairs used for qRT-PCR analysis of different genes are mentioned in S1, S2 and S3 Tables.

### Protein extraction and immunoblot analyses

Frozen root tissues were ground in liquid nitrogen and homogenized in 1 ml of ice-cold extraction buffer (100 mM HEPES-KOH pH 7.5, 5 mM EDTA, 5 mMEGTA, 10 mM DTT, 10 mM Na_3_VO_4_, 10 mM NaF, 50 mM β-glycerol phosphate, 1 mM PMSF, 10% glycerol and 7.5% PVPP). Extracts were centrifuged and the clear supernatant was recovered. For western 20 μg of extracted crude protein was fractionated on a 10% polyacrylamide gel containing 0.1% SDS. Proteins were transferred to a polyvinylidene difluoride (PVDF) membrane in a BIO-RAD semi-dry blot tank using transfer system according to manufacturer’s protocol for 1h. For immuno-detection of proteins, the membrane was blocked with 5% (w/v) skimmed milk in Tris-buffered saline (TBS) buffer for 2h, and subsequently incubated with primary antibody diluted in TBS-T buffer (TBS 0.1%, Tween 20) containing 3% (w/v) skimmed milk for 1.5 h at room temperature or at 4°C overnight. After washing in TBS-T the membrane was incubated with secondary antibody diluted in TBS-T containing 3% (w/v) skimmed milk at room temperature for 1.5 h. Following several washing steps, proteins were detected by incubating the membrane in freshly prepared chemiluminescent HRP substrate (Immobilon Western, Millipore Corporation,U.S.A.). Chemiluminescence was detected using HyperProcessor (Amersham Biosciences). Sources and dilutions of antibodies are listed in S4 Table.

### In Gel Kinase Assay

The in-gel kinase activity was carried out as described previously [30]. 20 μg of total protein was fractionated on a 10% polyacrylamide gel containing 0.1% SDS and 0.5 mg ml^-1^ bovine brain myelin basic protein (MBP) (Sigma Aldrich). After electrophoresis, the SDS from the gel was removed with buffer (25 mM Tris–HCl pH 7.5, 0.5 mM DTT, 5 mM Na_3_VO_4_, 0.1 mM NaF, 0.5 mg ml BSA, 0.1% Triton X 100) followed by renaturation in buffer (25 mM Tris–HCl pH 7.5, 0.5 mM DTT, 5 mM Na_3_VO_4_, 0.1 mM NaF) at 4°C overnight. MBP phosphorylation was performed by incubating the gel in 20 ml of reaction buffer (25 mM Tris HCl pH 7.5, 2 mM EGTA, 12 mM MgCl_2_, 1 mM DTT, 0.1 mM Na_3_VO_4_, 1 μM ATP and 50 μCi of γ^32^P-ATP (3000 Ci mmol-1) for 60 min at room temperature. The gel was washed three times with 5% TCA and 1% Sodium pyrophosphate and auto radiographed in phosphor imager (Typhoon, GE health care).

## Results

### High throughput assays using GiA roots software revealed various novel rice root phenotypic parameters

**Fig 1:**
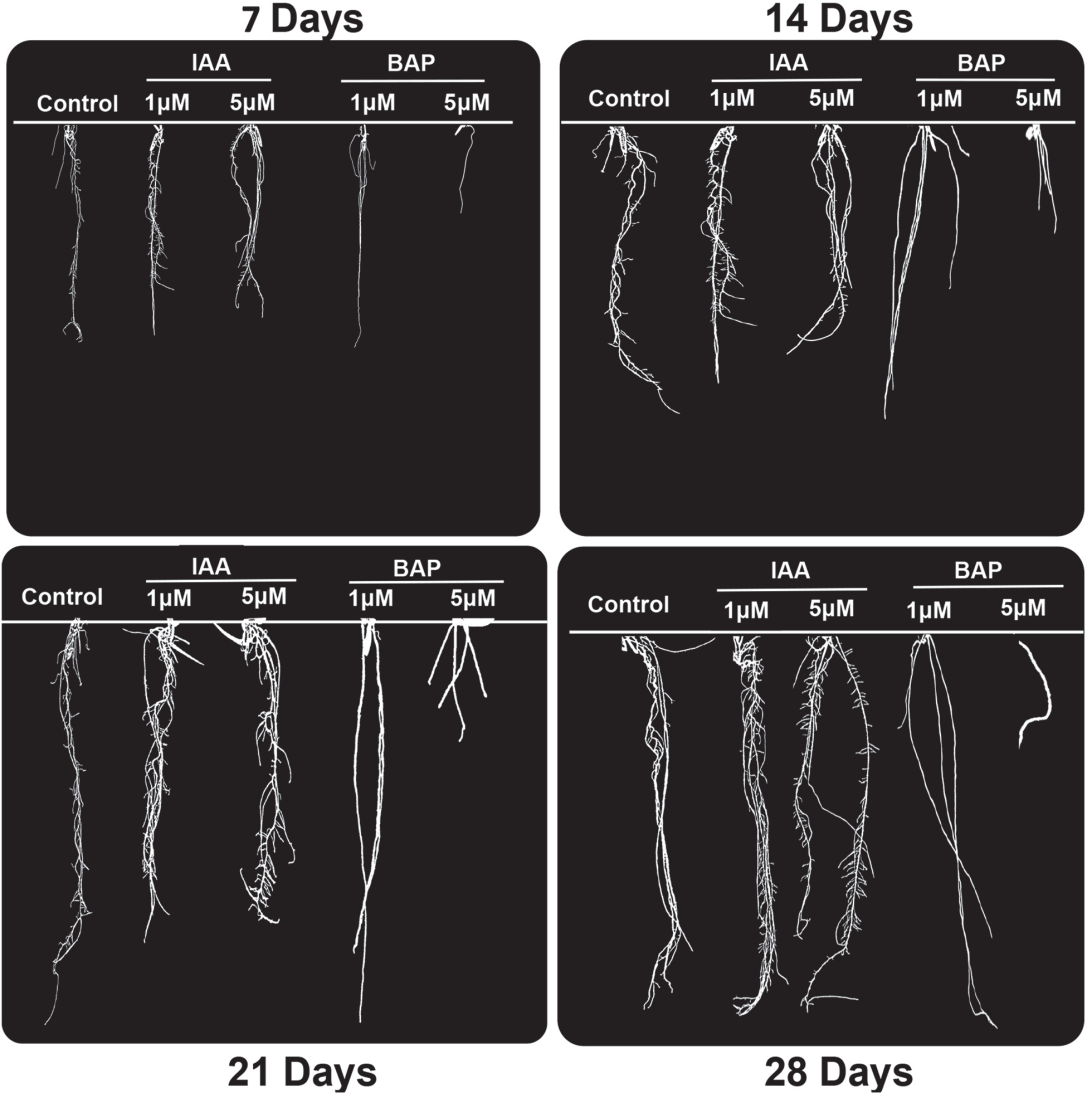
Novel phenotypic variability on auxin and cytokinin treatment revealed by GiA Roots Software framework. Annotated images by GiA roots software of 7 days, 14 days, 21 days and 28 days old rice seedling treated with 1, 5μM IAA and BAP in comparison with the normally grown seedlings.

**Fig 2:**
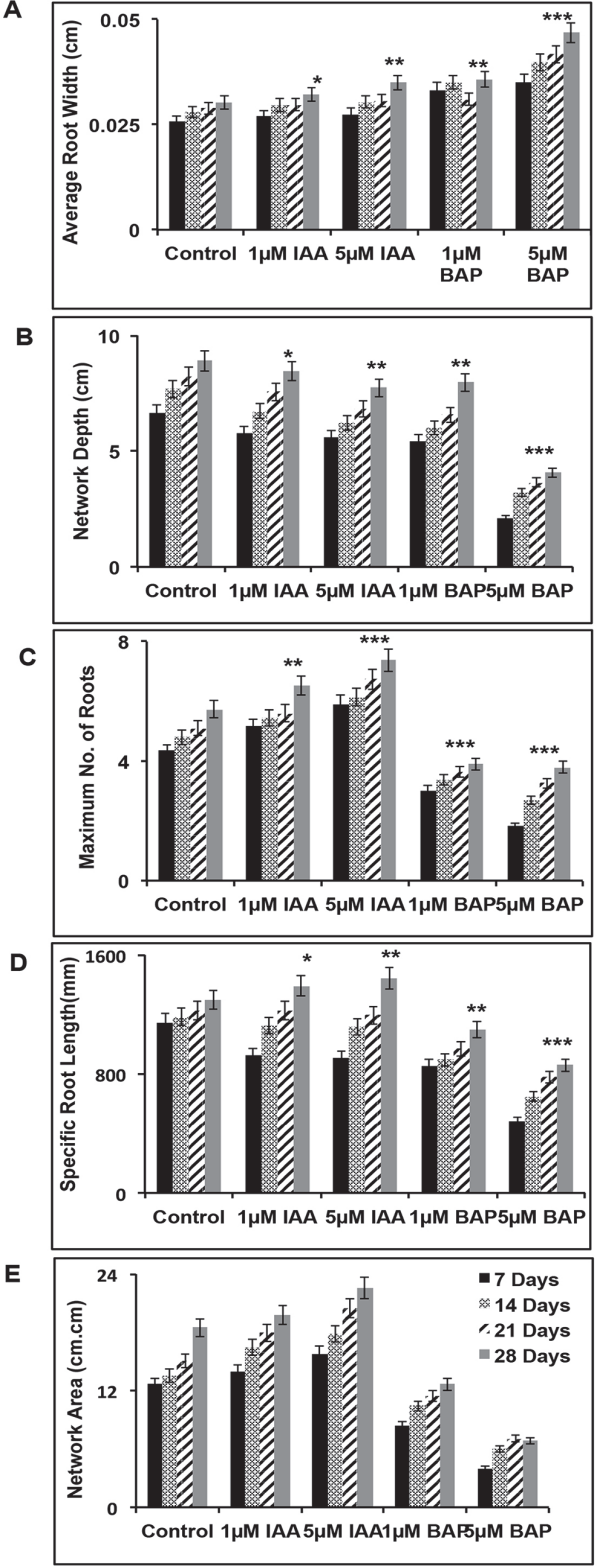
Phenotypic parameters show marked variability on auxin and cytokinin treatment. Graphical representation of root phenotypic parameters of untreated as well as 1, 5μM IAA and BAP treated 7, 14, 21 and 28 days rice seedlings. Average Root width (**A**), Network Depth (**B**), Maximum number of Roots (**C**), Specific Root Length (**D**) and Network Area (**E**) was analyzed using GiA roots software and plotted into bar graphs. Data shown is the average of three triplicates with error bars indicating standard deviation. The asterisks indicate a significant differences between Control vs. IAA treated and Control vs. BAP treated (***, P < 0.001), (*, P < 0.05) and (**, P < 0.01).

Estimation of Root system architecture (RSA) traits may give deeper insights into rice phenotype, which in turn may pave way in deciphering various complex nexus of root developmental aspects. Hence, in the present study, a large number of rice root system images were analyzed using GiA roots software. The annotated data resulted in generation of processed images of 7, 14, 21 and 28 days old rice roots of untreated, IAA and BAP treated seedlings (Fig 1). GiA roots software was also used for the elucidation of 19 root specific phenotypic traits out of which a few of the conclusive data have been plotted into histograms in Fig 2. A time course increase of average root width, network depth, maximum number of roots, specific root length and network area was observed in all rice seedlings. Marked increase in root width (Fig 2A) was observed in BAP treatment with a substantial increase at higher concentration. Higher concentration of IAA also resulted in increment of root width while lower concentration of the same was comparable to that of untreated seedlings. Network Depth (Fig 2B) was most pronounced in untreated rice seedlings while with a gradual concentration increase of the two treatments it deteriorated. Maximum extent of root branching (Fig 2C) was observed in IAA treatment, while in case of BAP treatment there was steep decline in root branching. The specific root length (Fig 2D) on IAA treatment was almost comparable to untreated samples while BAP treatment led to a significant decrease in specific root length. In comparison of untreated seedlings a slight increase in network area (Fig 2E) was observed in case of IAA treated seedlings while BAP treated seedlings were compact with decreased area coverage. The other 14 novel root phenotypic traits namely network bushiness, ellipse axis ratio, network length distribution, major ellipse axis, network width, median number of roots, minor ellipse axis, network convex area, network perimeter, network solidity, specific root length, network length, network volume and network width to depth ratio are represented graphically and documented in S1 Fig. Thus, from the above extensive phenotypic analysis it may be concluded that IAA treatment results in increment in maximum and median number of roots, network bushiness, specific root length, network convex area and network perimeter while BAP treatment results in increase in network width, network width to depth ratio, average root width and network solidity.

### Different members of MAPK, MAPKK and PIN gene family in rice are regulated during root development

**Fig 3:**
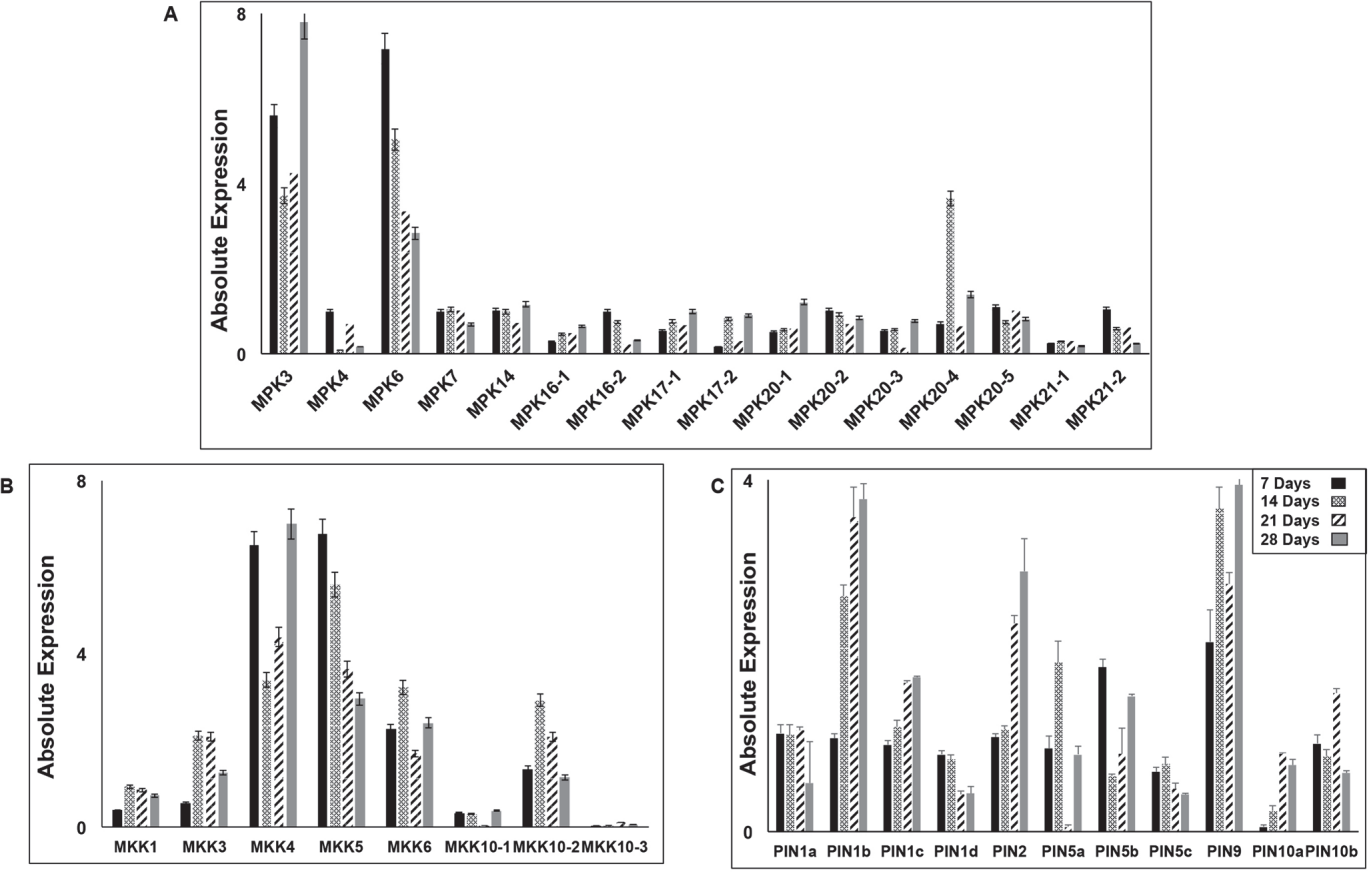
Differential transcript accumulation of all the components of MAPKs, MAPKKs and PINs. Absolute transcript levels of MAPKs (**A**), MAPKKs (**B**) and PIN (**C**) gene family members analyzed by qRT-PCR. Data shown is the average of three triplicates normalized to actin, with error bars indicating standard deviation.

After a comprehensive view of root architectural traits, study was carried to get a better insight at the molecular cues governing root development. qRT-PCR of 7, 14, 21 and 28 days old rice roots was undertaken to determine the absolute transcript levels of the complete family of MAPK, MAPKK and PIN genes (Fig 3). Among the 16 components of MAPK family (Fig 3A), *OsMPK3* and *OsMPK6* showed high transcript accumulation. *OsMPK3* showed a high expression profile at 7 days, got reduced on 14 and 21 days stage and increased by eight fold in 28 days root samples. *OsMPK6* showed a temporal decrease in transcript accumulation with highest expression at 7 days stage and lowest at 28 days. The other members of MAPK gene family did not show any major drastic accumulation or alteration in their transcript level at the studied time point of root growth except for *OsMPK20-4* that showed high expression only on 14 days stage. In case of 8 MAPKK gene members, depicted by absolute transcript accumulation graph in Fig 3B, *OsMKK4* and *OsMKK5* showed significant alteration of transcripts. *OsMKK4* showed high transcript accumulation at 7 and 28 days root stage while in case of *OsMKK5* high transcript abundance was observed at 7 and 14 days stage which declined in 21 and 28 days. Further, expression patterns of 11 *OsPIN* (Fig 3C) was also assayed which identified *OsPIN1b*, *OsPIN2* and *OsPIN9* as potential candidates for study due to their high and varied transcript profile.

### Auxin and Cytokinin lead to a closely inter-related regulation of the potential MAPK and PIN family components

**Fig 4:**
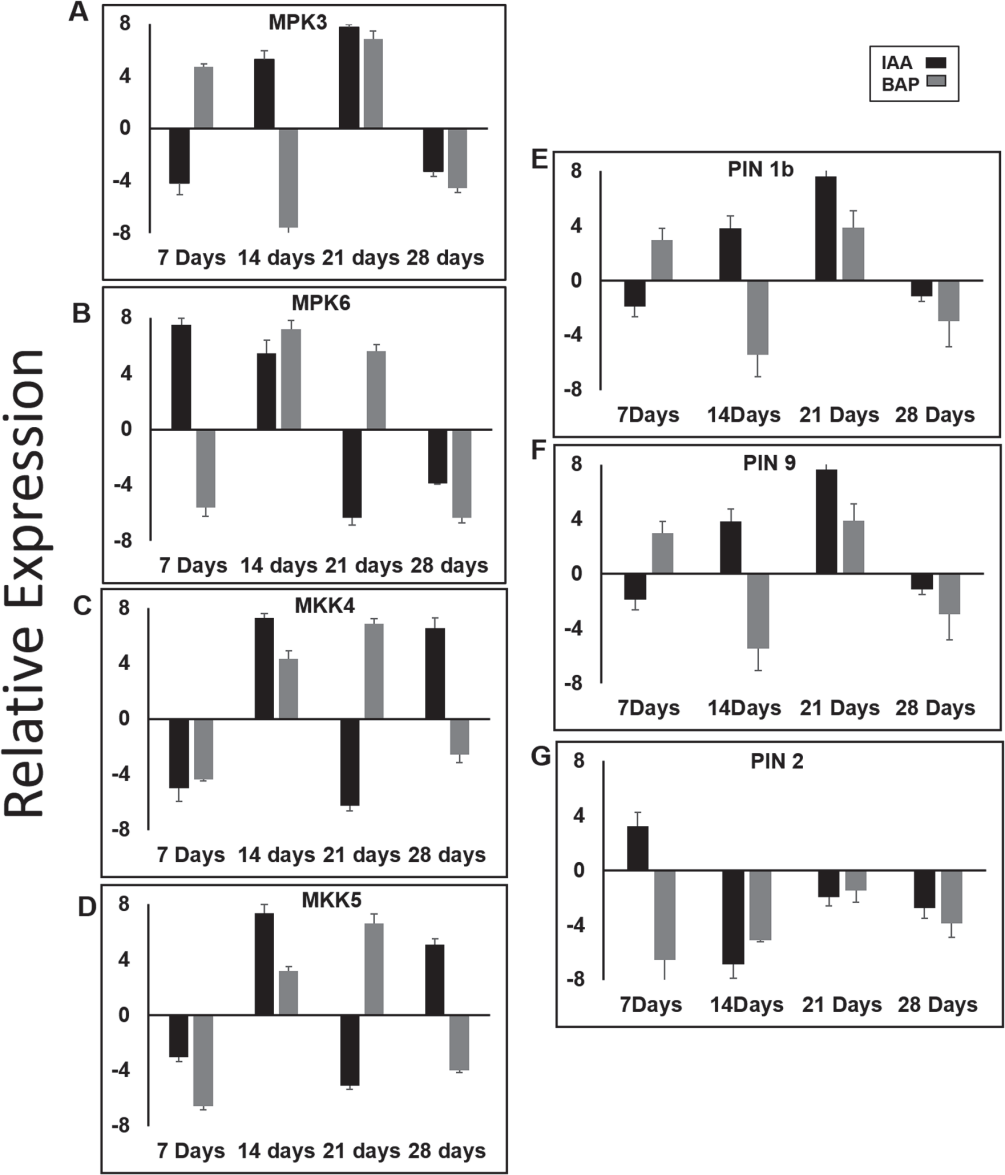
Expression of MAPK, MAPKK and PIN gene family members show intricate regulation on auxin and cytokinin treatment. Transcripts level profile of OsMPK3 (**A**), OsMPK6 (**B**), OsMKK4 (**C**), OsMKK5 (**D**), OsPIN1b (**E**) OsPIN9 (**F**) and OsPIN2 (**G**) in response to auxin and cytokinin by qRT-PCR. Seedlings of 7, 14, 21 and 28 days were subjected to 1μM auxin and 1μM cytokinin treatments in ½ MS media. Expression level of untreated samples was taken as the baseline and all values shown are respective to baseline. Error bars indicate standard deviation of three independent experiments.

With a basic knowledge of the role of auxin and cytokinin as being the major players mediating root development and knowing about the expression patterns of the various components of MAPK signaling cascade and PIN genes, it was imperative to explore the differential expression of these components on auxin and cytokinin treatment. qRT-PCR analysis of 7, 14, 21 and 28 days old rice roots was undertaken to assess the transcript levels of the complete family of MAPK, MAPKK and PIN genes this time after 1 μM of IAA and 1 μM of BAP treatment. The response of the genes to auxin and cytokinin was assayed in reference to the normalized absolute transcript levels. Of all the MAPK genes (S2 Fig), *OsMPK3* and *OsMPK6* showed substantial response to auxin and cytokinin treatment as is evident by high and varied accumulation of transcripts of the two genes. The initial response at 7 days stage of the two genes was completely contrasting (Fig 4A and 4B). *OsMPK3* was down regulated by IAA treatment and up- regulated by BAP treatment, a vice versa scenario was observed in case of *OsMPK6*. An interesting expression pattern was observed at 14 and 21 days where *OsMPK6* showed up-regulation in both treatments while, *OsMPK3* followed the same trend in 21 days old samples. At 14 days stage *OsMPK3* was up- regulated by 6 fold and down-regulated by almost 7.5 fold on IAA and BAP treatments, respectively. *OsMPK6* was down regulated by almost 7.5 fold and up- regulated by 6 fold on IAA and BAP treatments, respectively at 21 days stage. Paradoxically, there was a marked down regulation of both the genes at the fourth week stage. Higher down-regulation was observed on BAP treatment in both the cases. A comprehensive assay of transcript profile of all the members of *MAPKK* (S3 Fig), identified *OsMKK4* and *OsMKK5* as potential candidates responding to auxin and cytokinin treatment. As is elucidated in Fig 4C and 4D both the genes showed a concerted expression trend. At 7 days stage both IAA and BAP treatments lead to the down regulation of the two genes, while at 14 days stage both the genes were chronically up regulated after the two treatments. 21 days resulted in down- regulation of both the genes on IAA treatment and a sudden up-regulation on BAP treatment while a contrasting trend was observed at 28 days stage, with IAA treatment resulting in up regulation and BAP treatment in down- regulation of both the genes. Complete transcript profile of members of PIN gene family (S4 Fig) was also undertaken. Notably, *OsPIN5c* showed negligible modulation on auxin and cytokinin treatment and hence, its results are not included in the present study. *OsPIN1b*, *OsPIN2* and *OsPIN9* were the putative candidates, which responded to IAA and BAP treatments by significant transcript accumulation as is clearly documented in Fig 4E–4G. *OsPIN1b* and *OsPIN9* emerged as important members of PIN gene family showing a marked accumulation of transcripts on hormonal treatment. Both *OsPIN1b* and *OsPIN9* were up regulated with BAP treatment at 7 days stage while IAA led to up-regulation at 14, 21 days stage. Down-regulation of the two genes was observed at 28 days stage and incidentally on both the hormonal treatments. *OsPIN2* showed an almost four fold up regulation on IAA treatment at 7 days stage while, both IAA and BAP treatment at the other temporal points led to a marked down regulation.

### Protein levels of OsMPK3 and OsMPK6 corroborated with the transcript profile

**Fig 5:**
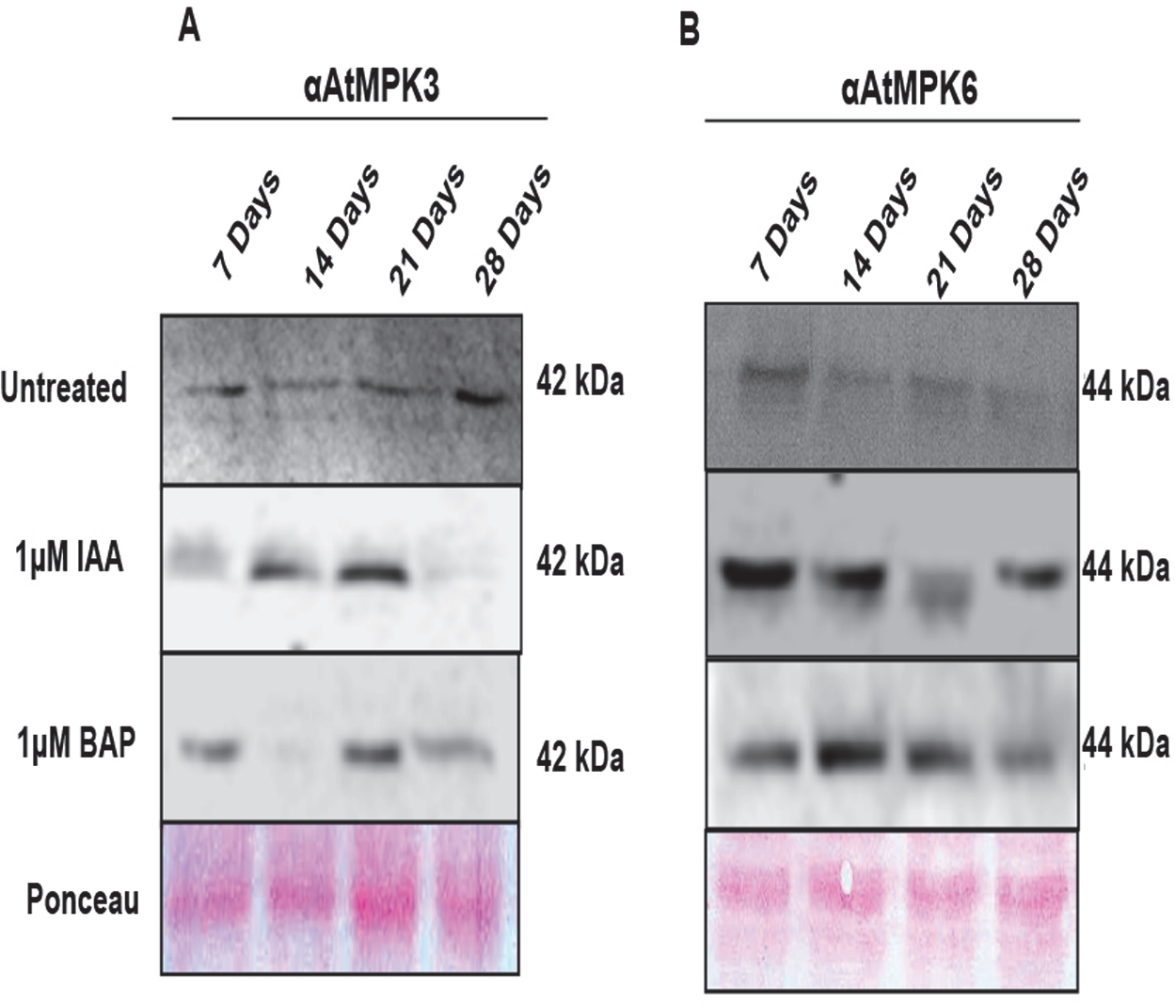
Protein levels of OsMPK3 and OsMPK6 co-relate with the respective transcript profile. Western blots showing the protein levels of OsMPK3 (**A**) and OsMPK6 (**B**) in 7, 14, 21 and 28 days old rice roots upon IAA (1μM) and BAP (1μM) treatments. Anti-AtMPK3 and Anti-AtMPK6 antibodies were used to assess the respective protein levels of OsMPK3 and OsMPK6.

It is evident from previous studies [19] that OsMPK3 and OsMPK6 show high homology with their *Arabidopsis* counterpart. Additionally, cross-reaction of *Arabidopsis* AtMPK3 and AtMPK6 antibodies with their respective orthologs in rice, OsMPK3 and OsMPK6 has been demonstrated earlier [18]. Hence, in the present study, western analysis with AtMPK3 and AtMPK6 specific antibodies were undertaken to assess the protein levels of OsMPK3 and OsMPK6. OsMPK3 protein level profile (Fig 5A) elucidated high protein level at 7 and 28 days stage in untreated tissues. On IAA treatment considerable protein levels were observed at 14 and 21 days stage while, BAP treatment lead to high protein levels at 7 and 21 days stage. As depicted in Fig 5B, in case of untreated root samples, OsMPK6 showed highest protein level at 7 days stage which declined till the fourth week. On IAA treatment high protein levels were observed at 7 days that decreased at 14 and 21 days stage and waning to a minimum at the 28 days stage. On BAP treatment high protein level of OsMPK6 was observed at 14 and 21 days stage while protein was accumulated to a lesser extent at 7 and 28 days stage.

### IAA and BAP induce juxtaposed activation of OsMPK3 and OsMPK6

**Fig 6:**
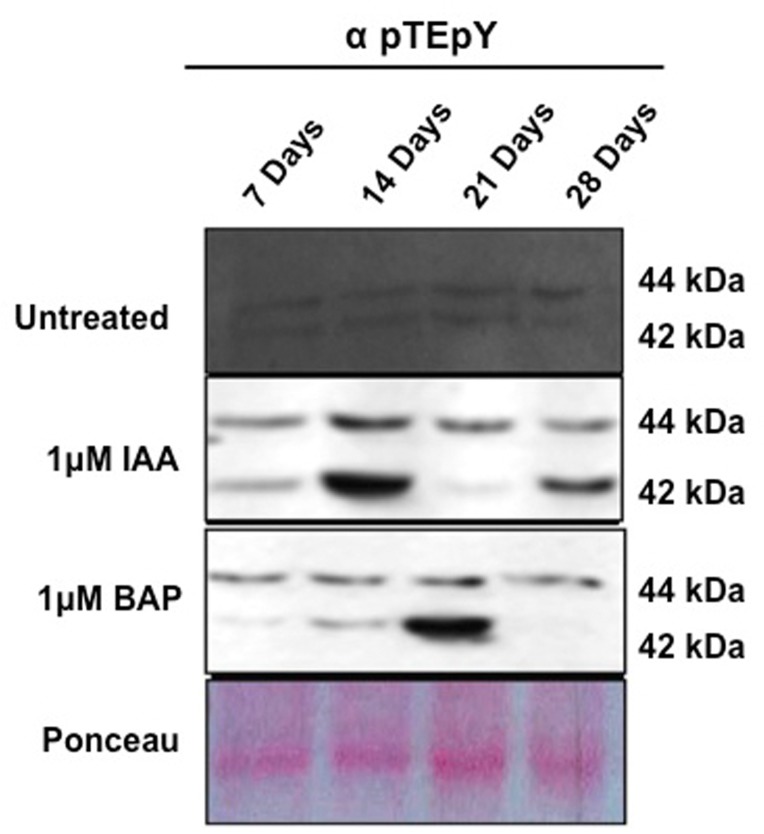
OsMPK3 and OsMPK6 show activation on auxin and cytokinin treatment in a contrasting manner. An immuno-kinase assay with pTEpY antibody, which specifically identifies the activated MAPK components, was performed. MAPK activity signals were observed at ~42, and ~44 kDa corresponding to the size of OsMPK3 and OsMPK6, respectively**.**

MAPKs are known to phosphorylate their downstream targets when activated through phosphorylation by the upstream MAPKK [16]. Therefore, protein activity of the potential MAPK members i.e. OsMPK3 and OsMPK6 was studied. An immuno-kinase assay was carried out with an anti phospho-threonine-glutamic acid- anti phospho-tyrosine antibody, pTEpY [31] that specifically recognizes the activated MAPK components (Fig 6). The immuno-kinase assay led to an interesting finding where OsMPK3 was activated temporally in a contrasting fashion on auxin and cytokinin treatment. Marked increase in OsMPK3 activity was observed at 2 week stage on IAA treatment while on BAP treatment negligible activation was observed. A similar trend was also observed at 7 and 28 days stage but to a lesser extent. While, a contrasting trend was observed on BAP treatment at 21 days stage where BAP treatment led to marked activation and IAA treatment diminished the activity. OsMPK6 showed high activation at 14 and 21 days stage on IAA treatment while in all the other samples almost a basal activation was reported. Similar results have been observed with an in-gel MBP phosphorylation assay (S5 Fig)

## Discussions

In the present study a large-scale assay of rice root phenotype as well as the effect of auxin and cytokinin at different temporal stages of root development was undertaken. This was a first high throughput experimental setup of its kind which tried to delineate almost 19 novel phenotypic characters, in particular, of the rice roots. There have been some reports [32–34] exploring quite a few root related aspects particularly in *Arabidopsis* but to date, a similar study in rice is missing. The approach adopted in the present study, was to delineate the signaling cascade (in particular MAPK network) governing the different temporal growth stages of rice root and then correlating it with cardinal phytohormones like auxin and cytokinin, rather than exploring the events of the bifurcated zones. Although, exploitation of signaling events governing the development of different root zones will elucidate a number of intricate mechanisms. The current approach will provide a more holistic and conclusive involvement of the MAPK signaling cascade. The phenomics data added a few parameters to the concept of the present study as well as established various unexplored traits about RSA post auxin and cytokinin treatment. A comprehensive exploration of the annotated root phenomics data could pave way for elucidation of new dimensions of root physiological processes.

Molecular elucidation of major signaling cascades and their respective components may help in comprehending various factors affecting different aspects of rice root development. Growing understanding of the subject indicates that auxin acts as a central node, and various other phytohormones in particular cytokinin communicate with it to regulate root development [4,5,32,35]. In addition, signaling pathways that involve other plant hormones also influence the role of these two phyto-hormones in roots [36]. This multilevel interaction involves various phytohormone biosynthesis genes, auxin transporter genes, and a conspicuous signaling network like MAPK cascade, as well as a few other secondary messengers [2]. Although, innumerable reports have provided evidence of the involvement of MAPK cascade in various developmental aspects [12] yet a complete confirmation of the established role of MAPK network in root development is lacking. A few reports have speculated the role of MAPK signaling network in roots particularly in *Arabidopsis* [20,21,23,37,38] other reports have also linked MAPK signaling cascade with auxin signaling [24,25,39–41]. In spite of all these studies, there has never been a concerted effort to characterize all the components of MAPK network and co-relating them with phytohormonal regulation in rice. OsMPK3 and OsMPK6 emerged as putative targets of the present study with high expression profile. Both MPK3 and MPK6 are widely explored members of MAPK network, involved in varied number of developmental processes like ovule, anther and stomatal patterning as well as development [12,42–45]. Earlier studies have established the role of AtMPK6 in root development, documenting its role in repression of primary and lateral root development [21] and its differential localization in cell division plane control and root development [20]. The involvement of MPK3 in root development is currently unexplored and the present study is the first report of its kind. Furthermore, OsMKK4/5 are well established upstream counterparts of OsMPK3/6 and activate the latter by phosphorylation [46,47]. The role of this particular module is well studied in stomatal patterning and development [43]. Here we report the plausible involvement of this particular module in root development. It is evident that auxin mounts its effect by differential polar transportation facilitated by efflux carriers namely PIN proteins. Cytokinin also regulates the expression pattern of PIN to administer an intricate control on auxin signaling [48]. Hence, gene expression studies of PIN family identified *OsPIN1b*, and *OsPIN9* as potential members among PIN genes. The absolute expression quantification of these PIN genes were in accordance with a recent tissue specific characterization study [6]. Our present work was different from this previous study, as it deciphered the temporal expression profile of these *OsPIN* genes at different temporal stages of root development. Results of this study led to a conclusive elucidation of PIN genes being differently regulated at various stages of rice root development.

Both *OsMPK3* and *OsMPK6* were responsive to auxin and cytokinin treatment. Auxin resulted in up-regulation of *OsMPK3* transcripts in later stages (14 and 21 days) while *OsMPK6* was up regulated at early (7 and 14 days) root development stage. It is likely that the two MAPK components are playing a synergistic role in auxin treatment. *OsMPK6* being responsive to auxin treatment in early development stage and *OsMPK3* taking over the function during later stages. Cytokinin caused up regulation of OsMPK6 at later stages (14 and 21days). Interestingly, *OsMPK3* transcript showed a mixed trend being up regulated at 7 and 21 days and down regulated at 14 and 28 days stage. *OsMKK4*/*5* showed a similar expression trend on the phytohormonal treatment, which reinstates the redundant nature of the two genes. The redundant nature of MKK4/5 is elucidated in stomatal development and patterning [43,45], the current observation may be regarded as an extrapolation of the previous report. The transcript profile of *OsMKK4*/*5* coincided with the root development stage dependent activity of OsMPK3, the downstream counterpart of the signaling module. Hence, the two genes may act in a concerted manner to activate the downstream signaling components, which may further lead to activation of various root development related genes.

The co-regulation of *OsPIN1b/OsPIN9* and *OsMPK3* are indicative of the two genes being regulated by the phytohormones in a concerted manner. Hence, OsPIN1b/OsPIN9 may be intricately regulated by OsMPK3 on phytohormonal elicitation further, mounting their role in root growth and development. Further, phosphorylation of PIN proteins leads to conformational variation, which causes functional diversity. This in turn results in perturbed polar auxin transport and altered root development. OsMPK3 and OsMPK6, at a few instances, act in a redundant manner and share many of the substrates [49], these PINs could very well be the target of both OsMPK3 and OsMPK6. The sequence analysis of OsPIN1b and OsPIN9 also revealed the presence of a MAPK target site. It would be interesting to explore the phosphorylation status of these PIN proteins with MAPK cascade components, in particular OsMPK3, which would further elucidate the complex network of the MAPK signaling module and its phytohormonal regulation during rice root developmental stages.

**Fig 7:**
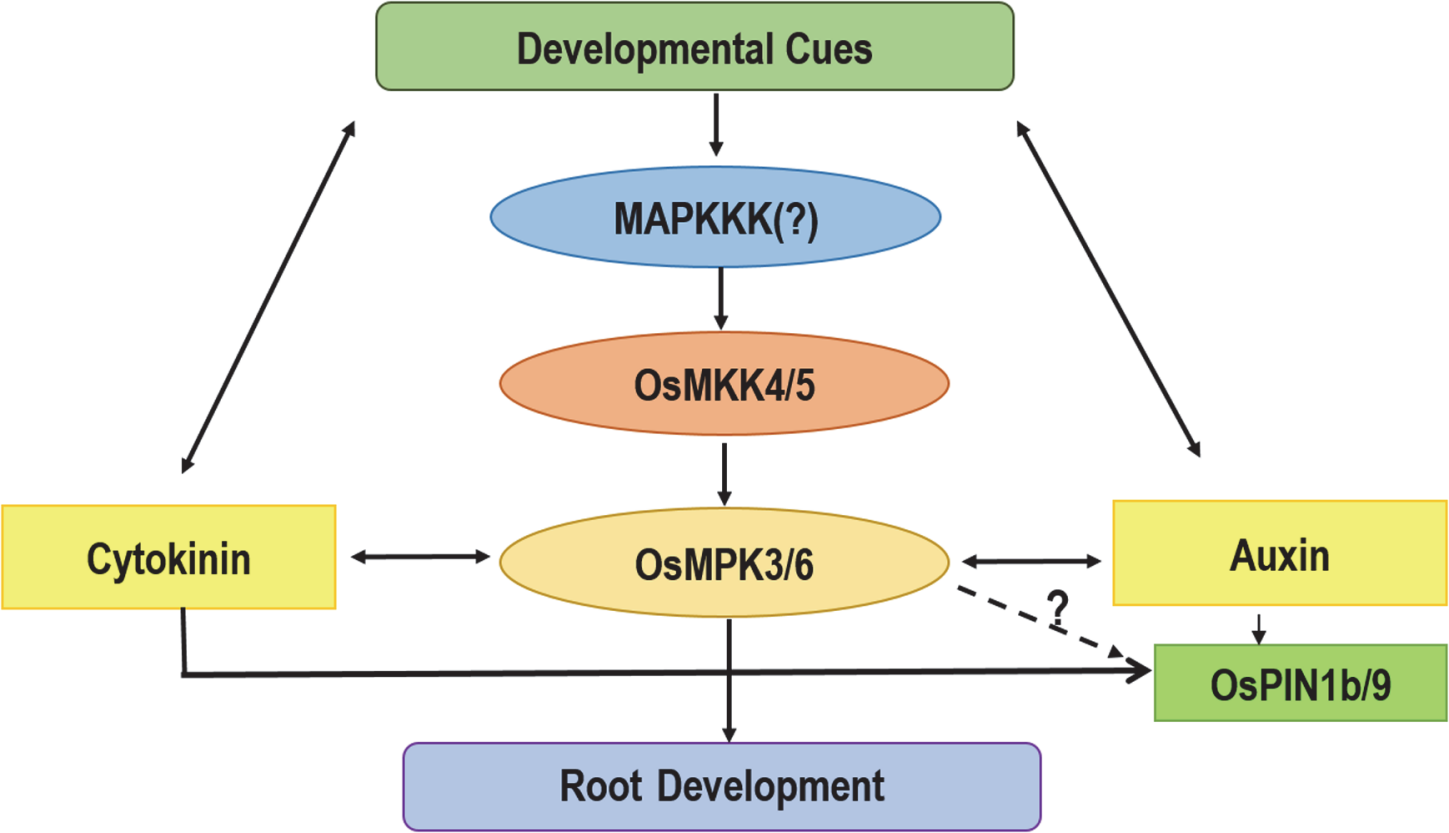
MAPK cascade and auxin-cytokinin interplay in root development. The testable model shows the involvement of the OsMKK4/5 and OsMPK3/6 module regulating OsPIN1b/9 and governing the root development phenomena in concert with auxin and cytokinin in rice.

Interestingly, the protein levels of the two potential MAPKs, namely OsMPK3 and OsMPK6 were in corroboration with their transcript profile. In subtle accordance with the transcript level, *OsMPK6* was responsive to auxin treatment in early development stage and *OsMPK3* proceeded over the function during later stages. Hence, both OsMPK3 and OsMPK6 may play crucial role at different temporal rice root development stage. Furthermore, the activity assay of the two MAPKs in question led to a very interesting finding. While, OsMPK6 was shown to be drastically activated on IAA and BAP treatment, OsMPK3 showed a unique and peculiar trend. It was observed to be antagonistically regulated by auxin and cytokinin. The above findings have been diagrammatically depicted into a testable model in Fig 7. These findings lead to an indicative conclusion of the involvement of OsMPK3 and OsMPK6 in rice root development which may act in concert or individually with the phytohormones at the specified time point and further lead to the modulation of transcript accumulation of OsPIN 1b/9 genes leading to various root developmental phenomena. The present study is a modest attempt to decipher the role of MAPK and hormonal interplay. We believe that the present study will be followed by a number of research undertakings which will further open a whole new realm of studies in the specific field of research.

## Conclusions

It is evident that auxin and cytokinin have antagonistic effects in root developmental processes [4,5,32,50] but no study has reported the involvement of MAPK signaling network in this intricate regulation. This study provides evidence that MAPK signaling network works in concert with auxin and cytokinin during various stages of rice root development. Further, the above study pinpoints at a nodal role of OsMPK3 and OsMPK6, which may act in concert or individually with the phytohormones at the specified time. These two pivotal components are in turn activated by upstream cascade component, OsMKK4/5. Furthermore, this module augments its role by affecting the polar transportation of PIN proteins via affecting the transcript accumulation of PIN genes namely OsPIN1b and OsPIN9.

## Supporting Information

S1 Fig Novel root phenotypic parameters in response to auxin and cytokinin treatment. Graphical representation of root phenotypic parameters of untreated as well as 1, 5μM IAA and BAP treated 1–4 week rice seedlings. All the root phenotypic parameters were analyzed using GiA roots software framework. Data shown is the average of three triplicates with error bars indicating standard deviation.
(PDF)

S2 Fig Expression pattern of MAPK cascade components in response to auxin and cytokinin treatment by qRT-PCR. On top of the left hand panel is relative expression pattern of a Group B MAPK i.e. *OsMPK4*, the next two graphs represents Group C MAPKs i.e. *OsMPK7* and *OsMPK14*, while all the others represent members of Group D MAPKs. Seedlings of 1–4 weeks were subjected to 1μM auxin and 1μM cytokinin treatments in ½ MS media. Expression level of untreated samples was taken as the baseline and all values shown are respective to baseline. Error bars indicate standard deviation of three independent experiments.
(PDF)

S3 Fig Relative expression patterns of MAPKK family members in response to auxin and cytokinin treatment by qRT-PCR. Seedlings of 1–4 weeks were subjected to 1μM auxin and 1μM cytokinin treatments in ½ MS media. Expression level of untreated samples was taken as the baseline and all values shown are respective to baseline. Error bars indicate standard deviation of three independent experiments.
(PDF)

S4 Fig Transcript profiling of OsPIN family genes in response to auxin and cytokinin treatment by qRT-PCR. Seedlings of 1–4 weeks were subjected to 1μM auxin and 1μM cytokinin treatments in ½ MS media. Expression level of untreated samples was taken as the baseline and all values shown are respective to baseline. Error bars indicate standard deviation of three independent experiments.
(PDF)

S5 Fig In-gel kinase assay using myelin basic protein (MBP) as substrate show MAPK activity signals at ~ 42, and ~44 kDa corresponding to the size of OsMPK3 and OsMPK6, respectively. (PDF)

S1 Table List of *MAPK* genes and primer pairs for qRT-PCR. (PDF)

S2 Table List of *MAPKK* genes and primer pairs for qRT-PCR. (PDF)

S3 Table List of *PIN* genes and primer pairs for qRT-PCR. (PDF)

S4 Table List of antibodies used for immunoblot analyses. (PDF)
