# Supplementary material for: Unraveling the Intricate Nexus of Molecular Mechanisms Governing Rice Root Development: OsMPK3/6 and Auxin-Cytokinin Interplay
Source: PLoS One. 2015 Apr 9;10(4):e0123620. doi: 10.1371/journal.pone.0123620 (PMC4391785; doi:10.1371/journal.pone.0123620)
Supplement: S1 Fig — Graphical representation of root phenotypic parameters of untreated as well as 1, 5μM IAA and BAP treated 1–4 week rice seedlings. All the root phenotypic parameters were analyzed using GiA roots software framework. Data shown is the average of three triplicates with error bars indicating standard deviation. (PDF) [file pone.0123620.s001.pdf]

Supporting Information

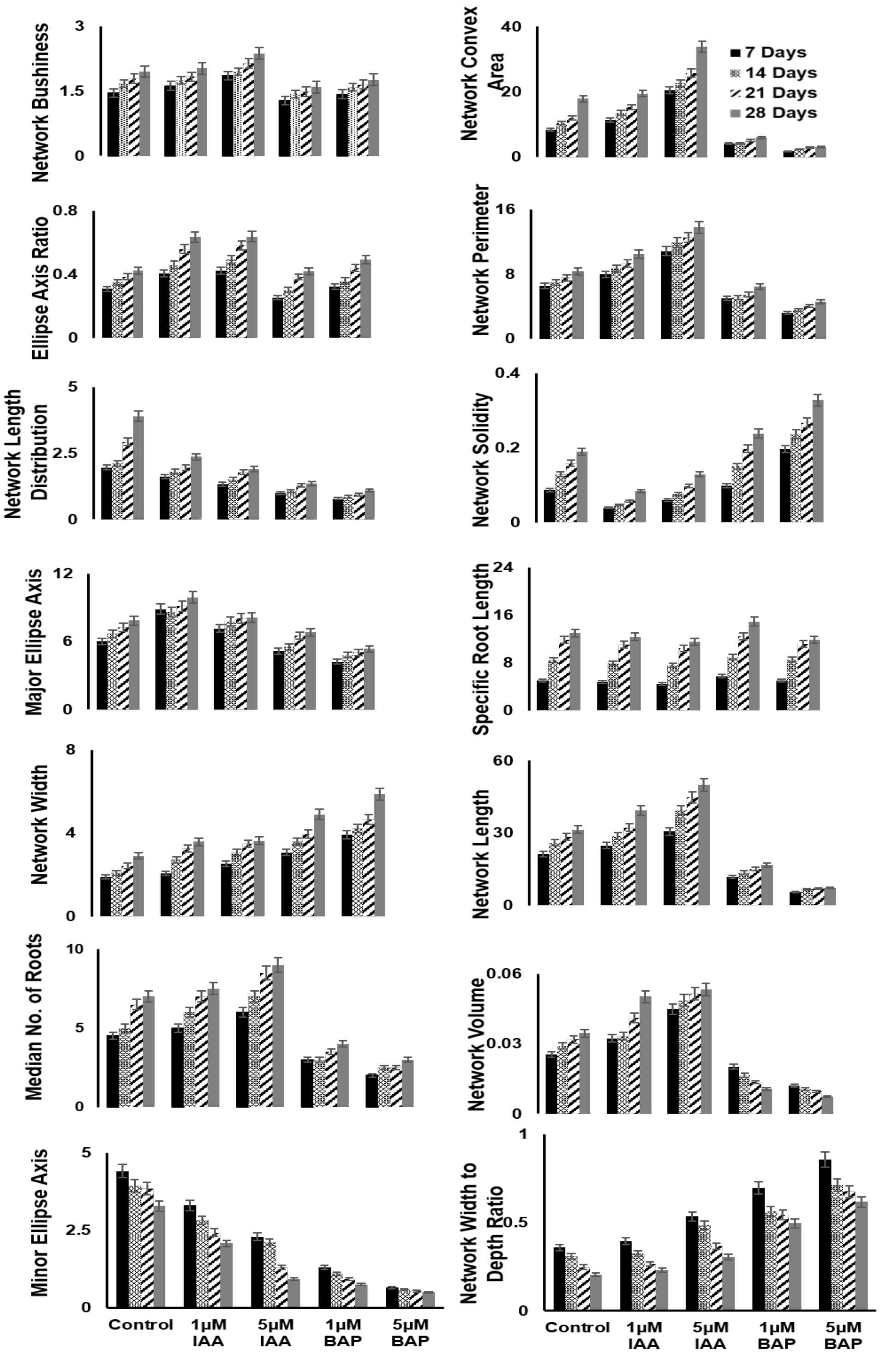

Figure S1: Novel root phenotypic parameters in response to auxin and cytokinin treatment. Graphical representation of root phenotypic parameters of untreated as well as 1, 5μM IAA and BAP treated 1-4 week rice seedlings. All the root phenotypic parameters were analysed using GiA roots software framework. Data shown is the average of three triplicates with error bars indicating standard deviation.
