# Supplementary material for: Unraveling the Intricate Nexus of Molecular Mechanisms Governing Rice Root Development: OsMPK3/6 and Auxin-Cytokinin Interplay
Source: PLoS One. 2015 Apr 9;10(4):e0123620. doi: 10.1371/journal.pone.0123620 (PMC4391785; doi:10.1371/journal.pone.0123620)
Supplement: S2 Fig — Expression level of untreated samples was taken as the baseline and all values shown are respective to baseline. Error bars indicate standard deviation of three independent experiments. (PDF) [file pone.0123620.s002.pdf]

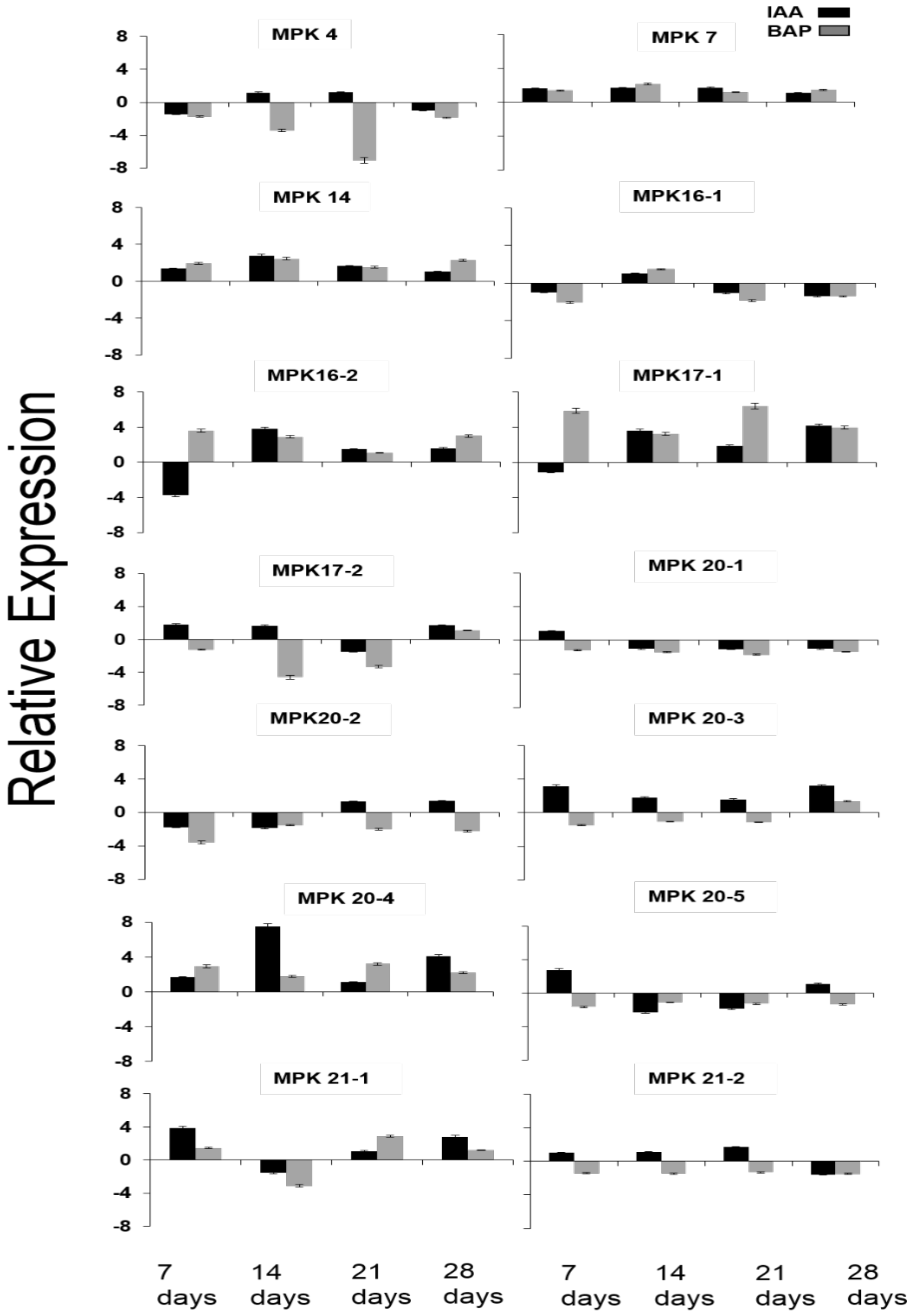

Figure S2: Expression pattern of MAPK cascade components in response to auxin and cytokinin treatment by qRT-PCR. On top of the left hand panel is relative expression pattern of a Group B MAPK i.e *OsMPK4*, the next two graphs represents Group C MAPKs i.e *OsMPK7* and *OsMPK14*, while all the others represent members of Group D MAPKs. Seedlings of 1 - 4 weeks were subjected to 1μM auxin and 1μM cytokinin treatments in ½ MS media. Expression level of untreated samples was taken as the baseline and all values shown are respective to baseline. Error bars indicate standard deviation of three independent experiments.
