# Supplementary material for: Unraveling the Intricate Nexus of Molecular Mechanisms Governing Rice Root Development: OsMPK3/6 and Auxin-Cytokinin Interplay
Source: PLoS One. 2015 Apr 9;10(4):e0123620. doi: 10.1371/journal.pone.0123620 (PMC4391785; doi:10.1371/journal.pone.0123620)
Supplement: S3 Fig — Seedlings of 1–4 weeks were subjected to 1μM auxin and 1μM cytokinin treatments in ½ MS media. Expression level of untreated samples was taken as the baseline and all values shown are respective to baseline. Error bars indicate standard deviation of three independent experiments. (PDF) [file pone.0123620.s003.pdf]

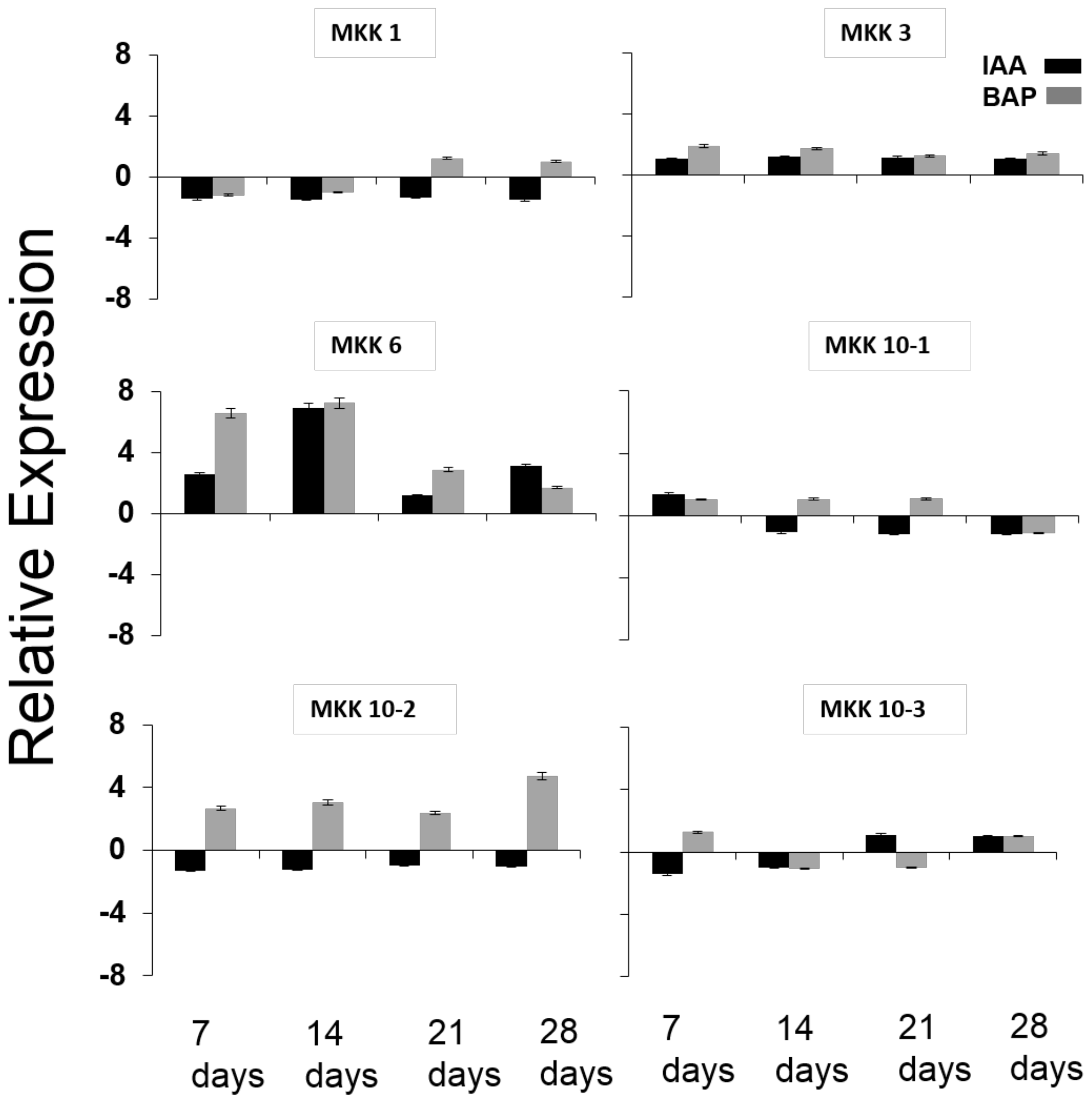

Figure S3: Relative expression patterns of MAPKK family members in response to auxin and cytokinin treatment by qRT-PCR. Seedlings of 1 - 4 weeks were subjected to 1µM auxin and 1µM cytokinin treatments in ½ MS media. Expression level of untreated samples was taken as the baseline and all values shown are respective to baseline. Error bars indicate standard deviation of three independent experiments.
