# Supplementary material for: Unraveling the Intricate Nexus of Molecular Mechanisms Governing Rice Root Development: OsMPK3/6 and Auxin-Cytokinin Interplay
Source: PLoS One. 2015 Apr 9;10(4):e0123620. doi: 10.1371/journal.pone.0123620 (PMC4391785; doi:10.1371/journal.pone.0123620)
Supplement: S5 Fig — (PDF) [file pone.0123620.s005.pdf]

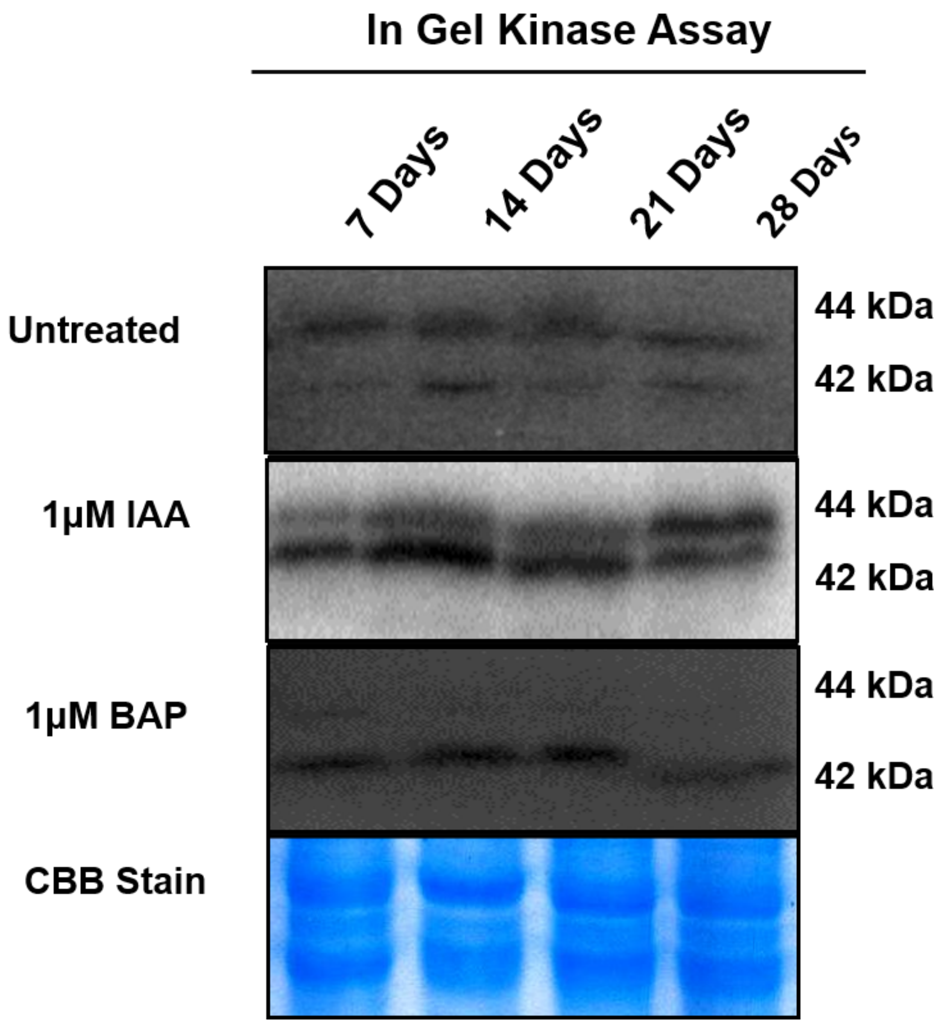

Figure S5: In-gel kinase assay using myelin basic protein (MBP) as substrate show MAPK activity signals at ~ 42, and ~44 kDa corresponding to the size of OsMPK3 and OsMPK6, respectively.
