# Supplementary material for: Unraveling the Intricate Nexus of Molecular Mechanisms Governing Rice Root Development: OsMPK3/6 and Auxin-Cytokinin Interplay
Source: PLoS One. 2015 Apr 9;10(4):e0123620. doi: 10.1371/journal.pone.0123620 (PMC4391785; doi:10.1371/journal.pone.0123620)
Supplement: S1 Table — (PDF) [file pone.0123620.s006.pdf]

Table S1 List of *MAPK* genes and primer pairs for qRT-PCR

**Serial No.**	**Gene**	**Length**	**Primer pairs**
1.	*OsMPK3*	19<br>17	5’-GCTCCAACCAAGAACTGTC-3’<br>5’-AGTCGCAGATCTTGAGG-3’
2.	*OsMPK4*	20<br>20	5’-CGAGGTCTCCTCCAAGTACG-3’<br>5’-GCGAAGCAGCTTGATTTCTC-3’
3.	*OsMPK6*	20<br>20	5’-AGGTCACCGCCAAGTACAAG-3’<br>5’-AGCAGCTTGATCTCCCTGAG-3’
4.	*OsMPK7*	17<br>17	5’-GCTCGCACAAACAACAC-3’<br>5’-GCCAAGAAGCTCAGCAA-3’
5.	*OsMPK14*	20<br>20	5’-TCCTGAGTTGCTCCTTTGCT-3’<br>5’-CGAGCTTTTGGGTTGTCAAT-3’
6.	*OsMPK16-1*	20<br>20	5’-CTTTGAGCATGTGTCCGATG-3’<br>5’-TTGGTGCAAATCAGACTCCA-3’
7.	*OsMPK16-2*	20<br>20	5’-TGGAGTCTGATTTGCACCAA-3’<br>5’-TGCAAGGCCAAAATCACATA-3’
8.	*OsMPK17-1*	20<br>20	5’-TCATCAGAGCGAACGATGAC-3’<br>5’-TGCAACATAATCCGTCCAAA-3’
9.	*OsMPK17-2*	18<br>19	5’-GCCTGCTAGAGCGTTTAC-3’<br>5’-CCTTTGTCAGTTTCCTTCG-3’
10.	*OsMPK20-1*	20<br>20	5’-TCGAGGAGGGATTTCAAAGA-3’<br>5’-CTTCAGGTCCCGGTGATAAA-3’
11.	*OsMPK20-2*	20<br>20	5’-GCTATGGGGTTGTGTGCTCT-3’<br>5’-ACAATGTCCGGATGCCTTAG-3’

12.	*OsMPK20-3*	20<br>20	5’-GATGCTCCGTGCTTTGAAAT-3’<br>5’-TGCAACATAATCGGTCCAGA-3’
13.	*OsMPK20-4*	17<br>20	5’-TTTCGCTCCCAAGGACT-3’<br>5’-CCCATTGCACTCAAATTCTC-3’
14.	*OsMPK20-5*	20<br>20	5’-AGAGAACCATCATGCCAACC-3’<br>5’-GTGTTTTCGGAGCCATTCAT-3’
15.	*OsMPK21-1*	20<br>20	5’-CGAGTGGCGATCAAGAAGAT-3’<br>5’-ATGTCCCTGAATTCCCTCCT-3’
16.	*OsMPK21-2*	20<br>20	5’-CCGGAGTTCTTCAGCGAGTA-3’<br>5’-AGGAGGCGGAGTAGCTTGAT-3’
