# Supplementary material for: Unraveling the Intricate Nexus of Molecular Mechanisms Governing Rice Root Development: OsMPK3/6 and Auxin-Cytokinin Interplay
Source: PLoS One. 2015 Apr 9;10(4):e0123620. doi: 10.1371/journal.pone.0123620 (PMC4391785; doi:10.1371/journal.pone.0123620)
Supplement: S2 Table — (PDF) [file pone.0123620.s007.pdf]

Table S2 List of *MAPKK* genes and primer pairs for qRT-PCR

**Serial No.**	**Gene**	**Length**	**Primer pairs**
1.	*OsMKK1*	20<br>20	5'-ACCATCGGCAAATTCCTGAC-3'<br>5'-GAACCAACTGCACGATTCCA-3'
2.	*OsMKK3*	20<br>20	5'-GTTGAATTCCAGGGTGCATT-3'<br>5'-TTCATGCAAGTAGCGCAAAC-3'
3.	*OsMKK4*	20<br>20	5'-GGACCATCGCCTACATGAGC-3'<br>5'-GGCGAGTCGGAGTAGCAAAT-3'
4.	*OsMKK5*	20<br>20	5'-CGGCCTCAGCATCCTAGAGT-3'<br>5'-ATAGCAGATGGCGCACATGA-3'
5.	*OsMKK6*	20<br>20	5'-TCCGAGGAAACTGCAGATGA-3'<br>5'-TTTGCGAACTGCCTCTTGAA-3'
6.	*OsMKK10-1*	20<br>19	5'-ACGTGTGGTCCCTGGGAGT-3'<br>5'-CCTCCTCCAGTCCTTCTCCA-3'
7.	*OsMKK10-2*	19<br>19	5'-ACCTCAAGCCGTCGAACCT-3'<br>5'-CTACCCCGAGGCTCCACAC-3'
8.	*OsMKK10-3*	18<br>20	5'-GCTGCTCCTCGAGCTGGT-3'<br>5'-AAGTCGGCGATCTTCACCTC-3'
