# Supplementary material for: Unraveling the Intricate Nexus of Molecular Mechanisms Governing Rice Root Development: OsMPK3/6 and Auxin-Cytokinin Interplay
Source: PLoS One. 2015 Apr 9;10(4):e0123620. doi: 10.1371/journal.pone.0123620 (PMC4391785; doi:10.1371/journal.pone.0123620)
Supplement: S3 Table — (PDF) [file pone.0123620.s008.pdf]

Table S3 List of *PIN* genes and primer pairs for qRT-PCR

Serial No.	Gene	Length	Primer pairs
1.	*OsPIN1a*	20<br>20	5'- GGGCTCCAGCTTCAACCACA -3'<br>5'- TGGAGGTCTTTGCCGTCCTC -3'
2.	*OsPIN1b*	19<br>20	5'- ACGCGTCCAAGCCCAAGTA -3'<br>5'- CTGTCCATGACGCCCCTGTT -3'
3.	*OsPIN1c*	20<br>20	5'- GCAGTCACACTCGCAGTCCA -3'<br>5'- GTCCTTCCTCTTCCCCACCA -3'
4.	*OsPIN1d*	20<br>20	5'- AGGTGAAGGAGGACGGCAAG -3'<br>5'- GAAGTCGGTGTGGTTGAAGC -3'
5.	*OsPIN2*	20<br>20	5'- GAGATCGAGGACGGGCTGAA -3'<br>5'- CTGATGAGCTTGCGCCACAC -3'
6.	*OsPIN5a*	20<br>19	5'- TCCGTCTTCCAGGCCATTGT -3'<br>5'- ACTTTCACCAGCGCCCACA -3'
7.	*OsPIN5b*	20<br>20	5'- TCCTGCACCTCGCCATCATA -3'<br>5'- CAAAGCCCAGAACCGCGTAA -3'
8.	*OsPIN5c*	20<br>20	5'- ACCCCTTCGCCCTCAGCTAC -3'<br>5'- CGACGAGCGTGTTGTTCAGC -3'
9.	*OsPIN9*	20<br>20	5'- GCTTCCTTGGCCTCATCTGG -3'<br>5'- GCATCACAACCGGGCCTATC -3'
10.	*OsPIN10a*	20<br>20	5'- ACGGCTCAGGCAAAGAGCAC -3'<br>5'- GCTCGACCCCATCTTCGTCA -3'
11.	*OsPIN10b*	20<br>20	5'- CGCTGTACGTGGCGATGATG -3'<br>5'- GACGAAGCGGAGGTTCATGG -3'
