# Supplementary material for: Unraveling the Intricate Nexus of Molecular Mechanisms Governing Rice Root Development: OsMPK3/6 and Auxin-Cytokinin Interplay
Source: PLoS One. 2015 Apr 9;10(4):e0123620. doi: 10.1371/journal.pone.0123620 (PMC4391785; doi:10.1371/journal.pone.0123620)
Supplement: S4 Table — (PDF) [file pone.0123620.s009.pdf]

Table S4 List of antibodies used for immunoblot analyses

**Name of Antibody**	**Manufacturer**	**Dilution**
Rabbit polyclonal anti P44/42MAPK (T202/Y204) pTEpY	CELL SIGNALING Technology	1:7,500
Rabbit polyclonal anti AtMPK6	SIGMA	1:10,000
Rabbit polyclonal anti AtMPK3	SIGMA	1:10,000
Horse Radish Peroxidase (HRP) coupled anti-rabbit IgGs	PIERCE	1:5,00
